# Tissue-Specific Antioxidant Activities of Germinated Seeds in Lentil Cultivars during Thermal Processing

**DOI:** 10.3390/antiox12030670

**Published:** 2023-03-08

**Authors:** Ji Hye Kim, Shucheng Duan, You Rang Park, Seok Hyun Eom

**Affiliations:** 1 Graduate School of Green-Bio Science, College of Life Sciences, Kyung Hee University, Yongin 17104, Republic of Korea; 2 Department of Smart Farm Science, College of Life Sciences, Kyung Hee University, Yongin 17104, Republic of Korea

**Keywords:** lentil, germination, thermal processing, antioxidant activity, phytochemicals

## Abstract

Nongerminated seeds (NGS) and germinated seeds (GS) of lentils are regularly eaten after thermal processing. However, the effect of these high temperatures on the beneficial antioxidants present in seeds is unknown. This study examined the effects of thermal processing on the color, polyphenol content, and antioxidant activity (AA) of the seeds of three different cultivars of lentils, including two with seed coats, French green (FG) and Lentil green (LG), and one without a seed coat, Lentil red (LR). Regardless of the cultivars and processing temperatures, the GS tended to be clearer and less yellow than the NGS. The GS of the FG and LG showed lower levels of total phenolic content, major flavonoid content (kaempferol, luteolin, and myricetin), and AA than the NGS. On the other hand, the LR displayed the opposite trend, with the above indicators being higher in the GS than in the NGS. As the values in the germinated endosperm tended to increase, it was concluded that the decrease in AA in the FG and LG was caused by the reduction in antioxidants in the seed coat. Although the temperature had nonsignificant effects on the majority of the antioxidants in the NGS and GS of different lentil cultivars, an 80 °C treatment yielded the highest value of AA in the GS of FG and LG. The results of a correlation coefficient analysis demonstrated the significance of the content of kaempferol, total flavonoids, and total phenolics examined for this experiment as contributors to AA in lentil tissues.

## 1. Introduction

Lentil (*Lens culinaris*) is a commonly grown legume and is considered to be one of the top five healthiest foods in the world. Lentil comprises excellent nutritional properties, such as low fat, high protein, and high fiber contents [1,2,3]. However, the routine use of lentils is limited due to the presence of antinutritional factors, such as phytic acid, condensed tannin, lectins, and saponins [4,5,6].

Germination in legumes is an excellent way to reduce the quantity of nonnutritive compounds and increase the quantity of useful compounds, such as secondary metabolites, readily accessible carbohydrates, dietary fiber, and amino acids [7,8,9,10,11]. Increased contents of proteins, free amino acids, and vitamins have been reported in germinated lentils [12,13,14]. Aguilera et al. [15] suggested that the germination of lentil increases the melatonin content, which contributes to antioxidant activity (AA). It has also been reported that trypsin inhibitors, phytate, α-galactosides, and saponins were significantly lower in lentil sprouts than in raw seeds [13,16]. Importantly, it has been extensively observed that the germination of legumes can increase the concentration of health-promoting substances, such as polyphenols, which possess various bioactivities, such as antioxidant, anti-inflammatory, and cholesterol-lowering effects [11,17].

Lentil seeds are consumed as a dietary supplement in many countries and are either cooked with rice or eaten after boiling. Lentils can also be consumed in the form of sprouts. Sprouts are usually eaten directly as a salad or after bleaching. Meanwhile, as a nutritional supplement, germinated lentil flour has attracted significant attention in the food industry for improving the nutritional quality of products [18,19,20]. Thus, heat treatment is closely related to the daily consumption of lentils. The reduction in antinutrients in legumes, such as lentils, following thermal processing is widely recognized [4,21,22]. However, it remains unclear how thermal treatments affect the content of the health-beneficial compounds in germinated lentil seeds.

Therefore, the aim of this study was to investigate the effects of thermal processing on the color, total phenolic compounds (TPC), total flavonoid content (TFC), major flavonoids, and antioxidant activity (AA) of the nongerminated seeds (NGS) and germinated seeds (GS) of three commonly consumed cultivars of lentil in Korea.

## 2. Materials and Methods

### 2.1. Chemicals and Materials

All of the solvents and reagents used in this study were of analytical grade. We utilized three varieties of lentil seeds, including two with seed coats, i.e., French green (FG) whole seeds and Lentil green (LG) whole seeds, and one without a seed coat, i.e., Lentil red (LR) seeds. These seeds were purchased from Asia Seed Co., Ltd. (Seoul, Korea).

### 2.2. Sample Processing

Seed germination was performed as described by Atudorei et al. [23] with some modifications. In brief, the seeds were soaked in water for 8 h and then allowed to germinate on wet tissue paper for 40 h in the dark at 26 °C and 80% humidity (the length of GS was around 15 mm). Both the NGS and GS were washed with distilled water and dried on a paper towel. In experiment 1, the seed coats and endosperms were separated from both the NGS and GS for further analysis. The seeds were dried completely at 30 °C. In experiment 2, using a dry machine (Koencon Co., Ltd., Hanam, Korea), the seeds were dried at 60 °C, 80 °C, and 100 °C for 5, 4, and 3 days, respectively, until the water content was below 10%. All of the dried samples were pulverized using a commercial mixer and sieved with a 100-mesh size. The completely dried samples were used as the control.

### 2.3. Measurement of Color Variation

The RGB values for each sample were obtained using a color analyzer (Lutron Electronics, Inc., Coopersburg, PA, USA). The values of L* (lightness/darkness), a* (green/red), and b* (blue/yellow) were obtained by converting the RGB values using the OpenRGB software (version 2.30.10125, Logicol S.r.l., Trieste, Italy).

### 2.4. Sample Extraction

For the analysis of the total phenolic compounds, total flavonoid content, and antioxidant activities in the samples, 80 mg of lentil powder was extracted from each sample with 4 mL of 80% methanol (*v*/*v*) at room temperature for 12 h under shaking at 120 rpm. The supernatant was obtained after centrifugation at 12,000× *g* at 24 °C for 10 min. The supernatant was filtered through a 0.45 µm membrane filter and the filtrate was then stored at 4 °C until further analysis.

For the high performance liquid chromatography (HPLC) analysis of the individual polyphenols, 100 mg of powder was extracted at 80 °C for 2 h in 1.2 mL of 50% methanol (*v*/*v*) containing 1.2 M HCl. The supernatant was obtained after centrifugation at 12,000× *g* at 24 °C for 10 min. After being filtered through a 0.45 µm membrane filter, the solution was stored at 4 °C until the HPLC analysis.

### 2.5. Measurement of TPC and TFC

The TPC was determined using the Folin–Ciocalteu method, as described by Duan et al. [24], with some modifications. Briefly, 50 µL of the sample extract or standard were mixed to 650 µL of distilled water in a 2 mL tube. Then, 50 µL of Folin–Ciocalteu’s phenol reagent was added and vortexed immediately. After 6 min, 500 µL of 7% Na_2_CO_3_ was added and incubated for 90 min at room temperature. The absorbance of the solution was measured at 750 mm using a spectrophotometer. The TPC was expressed in mg of gallic acid equivalent (GAE)/g dry weight.

The TFC was measured by following the method of Lim et al. [25] with some modifications. In brief, 100 µL of extract or standard was added to a 2 mL tube containing 640 µL of distilled water. Then, 30 µL of 5% NaNO_2_ was added. After waiting for 5 min, 30 µL of 10% AlCl_3_ was added and mixed well. After 1 min, 200 µL of 1 M NaOH was added, and readings were taken at 510 nm using a spectrophotometer. The quantity of TFC was defined as mg of catechin equivalent (CE)/g dry weight.

### 2.6. HPLC Analysis

The Waters 2695 Alliance HPLC (Waters Inc., Milford, MA, USA) equipped with a prontosil 120-5-C18-SH 5 m (4.6 250 mm, Bischoff, Leonberg, Germany) column was used to perform the HPLC analysis. The mobile phases were solvent A (0.1% formic acid in water) and solvent B (0.1% formic acid in acetonitrile) with a linear gradient as follows: 0–5 min, 15–20% B; 5–15 min, 20–30% B; 15–20 min, 30–35% B; 15–20 min, 30–35% B; 20–25 min, 35–80% B; 25–34 min, 80% B; 34–36 min, 80–15% B; 36–40 min, 15% B. The injection volume of the samples was 10 µL, and the wavelength for the flavonoids was set at 360 nm. The quantitative analysis of the flavonoids was calculated by the standard compound kaempferol.

### 2.7. Measurement of Antioxidant Activity

Both the 2,2-diphenyl-1-picrylhydrazyl (DPPH) radical scavenging ability and the 2,2′-azino-bis (3-ethylbenzothiazoline-6-sulfonic acid) (ABTS) radical scavenging ability were used to assess the antioxidant activities of the samples. These assays were performed as described by Lim et al. [26] and Duan et al. [27], respectively. The radical scavenging activity of the samples was expressed as mg of vitamin C equivalent (VCE)/g dry weight.

### 2.8. Statistical Analysis

All the treatments were performed in triplicates. All of the results were expressed as the mean ± standard error (n = 3). The data were analyzed statistically using the SAS software (Enterprise Guide 7.1 version; SAS Institute Inc., Cary, NC, USA). An ANOVA followed by Tukey’s honestly significant difference (HSD) test were performed at a level of *p* < 0.05. Correlation analysis was carried out by calculating the Pearson’s correlation coefficients.

## 3. Results and Discussion

### 3.1. Morphology and Color Variation

**Figure 1:**
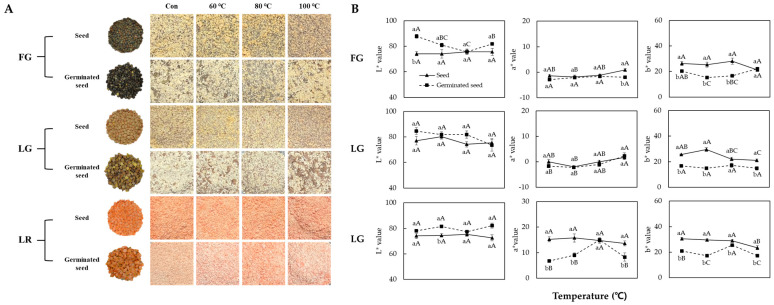
Seed morphology and powder color (**A**) of seed (nongerminated seed, NGS) and germinated seed (GS) in lentils (FG, LG, and LR) and CIE−color change of the powder (**B**) during thermal processing. Different lowercase letters indicate the significant differences (Tukey’s studentized test at *p* < 0.05) between NGS and GS, and capital letters indicates the significant differences (Tukey’s studentized test at *p* < 0.05) between lentil cultivars treated with different temperatures.

Figure 1A depicts the morphological changes caused by the germination process and thermal treatment in the NGS and GS of three different cultivars of lentil. Regarding the germination process, it was observed that the GS exhibited a darker surface color than the NGS, regardless of whether a seed coat was present or not. Similar patterns were observed in all of the varieties of lentil after thermal processing, regardless of the temperatures used. The GS had a brighter color and were less yellowish or reddish than the NGS. These observations support the CIE-color value variations in the NGS and GS of different cultivars of lentil during thermal processing (Figure 1B). Germination enhanced the L* value of all the lentil seeds, especially for the FG. However, the germination process decreased the a* and b* values. The L* value represented the perception of lightness, whereas a* and b* represented the perceived ratios of greenness/redness and blueness/yellowish, respectively [28]. The effects of the heat treatment on the color of the lentil seeds of different cultivars were not noticeable, and the values of L*, a*, and b* varied only slightly. However, the L* values in the GS of FG and LG showed downward tendencies as they were treated at higher temperatures. Interestingly, the L* value changes in the GS of the LR during thermal processing were not noticeable, whereas the a* value increased sharply when the seed was treated at 80 °C. The color of FG and LG came from the pigments in both the seed coat and endosperm, whereas the color of LR only came from the endosperm. The color differences in the NGS and GS of all three distinct cultivars of lentil based on either the germination process or thermal treatment may be explained by both enzymatic (enzymatic browning) and nonenzymatic (Maillard reaction or caramelization) processes that affect the pigments [25,28].

### 3.2. Changes in Total Phenolic Content 

**Figure 2:**
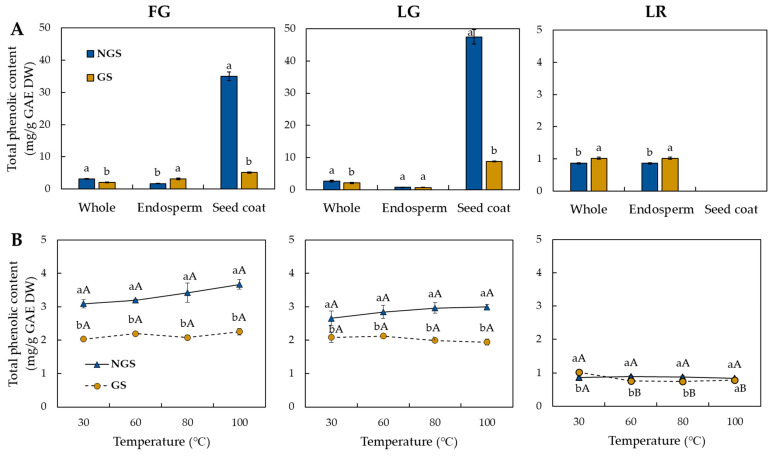
Changes in TPC of (**A**) different cultivars of lentil whole seeds, seed coat, and endosperm, (**B**) whole NGS and GS during different thermal treatment. Different lowercase letters indicate the significant differences (Tukey’s studentized test at *p* < 0.05) between NGS and GS, and capital letters indicates the significant differences (Tukey’s studentized test at *p* < 0.05) between lentil cultivars treated with different temperatures.

Figure 2A describes the TPC changes in the seed coat and endosperm of the NGS and GS of the different lentil cultivars. For whole lentil, the GS of FG (2.03 mg/g GAE DW) and LG (2.08 mg/g GAE DW) exhibited significantly decreased TPC compared with the NGS (3.09, 2.65 mg/g GAE DW for FG and LG, respectively), whereas the GS of LR (1.02 mg/g GAE DW) showed significantly increased TPC compared with the NGS (0.86 mg/g GAE DW). It has been shown that germination increases the TPC in sprouted seeds of several plants, including sunflower, radish, mung bean, and soybean [9,10,29,30]. However, germination has been reported to reduce the TPC content in lentils [31], similar to the FG and LG varieties but contrary to the LR variety (Figure 2A). Thus, it is important to choose a suitable variety when consuming lentil sprouts to obtain more TPC. FG and LG have a seed coat, whereas LR does not. Considering that the germination process may have different effects on the seed coat and endosperm of different lentil cultivars, the seed coat and endosperm from the FG and LG were separated and their TPC was analyzed. In Figure 2A, it can be observed that the TPC of the seed coat of the LG NGS (47.44 mg/g GAE DW) was higher than that of the FG seeds (35.02 mg/g GAE DW), although both were considerably reduced during germination. This decrease can be explained by the release of water-soluble compounds in the seed coat during seed germination. For the endosperm, the TPC was significantly increased in the FG, whereas it was stable in the LG. The breaking of seed dormancy and additional stimulation of enzyme activity that hydrolyze numerous components, including polyphenols, can serve as a basis for explaining the rise in the TPC in the lentil endosperm during germination [32,33]. In addition, the variation of the TPC in the germinated lentil endosperms depends on the cultivars. Therefore, it can be concluded that the release of polyphenols in the seed coats may be responsible for the significant decrease in the TPC in the germinated lentil seeds.

The effects of thermal processing on the TPC in the NGS and GS of the whole lentils are shown in Figure 2B. Thermal processing did not significantly affect the TPC in either the NGS or GS of the whole lentil, except for the germinated LR. High temperature (>60 °C) treatments considerably reduced the contents of LR GS, which exhibited higher TPC than the LG NGS. GS showed higher water content than the seeds, which may potentially accelerate the thermal energy transfer, leading to the degradation of thermal sensitive polyphenols, such as phenolic acids [28]. Considering the different seed compositions of the lentil cultivars, the presence of hard and strong seed coats in FG and LG may potentially reduce the heat transfer efficiency. According to these findings, phytochemicals in the NGS and GS of various cultivars of lentil have varying thermal stabilities.

### 3.3. Changes in Flavonoid Composition and Content

#### 3.3.1. Changes of Total Flavonoid Content

**Figure 3:**
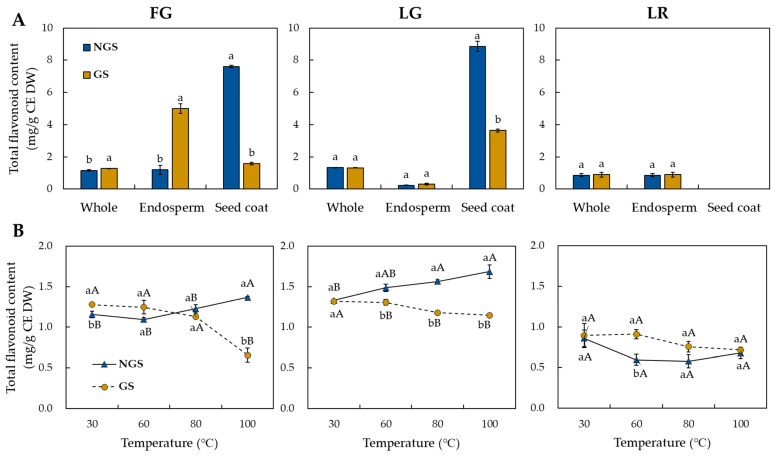
Changes in TFC of (**A**) different cultivars of lentil NGS and GS in whole, seed coat, and endosperm, (**B**) whole NGS and GS during different thermal treatment. Different lowercase letters indicate the significant differences (Tukey’s studentized test at *p* < 0.05) between NGS and GS, and capital letters indicates the significant differences (Tukey’s studentized test at *p* < 0.05) between lentil cultivars treated with different temperatures.

The TFC changes in the different cultivars of lentil, either during germination or after thermal processing, are shown in Figure 3. The effects of germination on the TFC varied across the lentil cultivars (Figure 3A). The TFC was substantially higher in the FG GS than in the FG NGS. Further analysis confirmed that the significantly increased TFC in the germinated endosperm (5 mg/g CE DW) might be responsible for a decrease in the TFC of the seed coat. For LG and LR, there were no appreciable changes in the TFC in the endosperm of the seeds or in the GS. In addition, the germination process significantly decreased the TFC in the seed coat of LG, which was similar to the results observed in the FG. These results suggested that the response of the TFC to germination varied depending on the lentil cultivars. Considering the ratio of endosperm to seed coat in lentils, it is recommended to consume the germinated endosperm of FG to obtain more TFC.

The effects of thermal processing on the NGS and GS are shown in Figure 3B. In the NGS from the lentil cultivars with a seed coat (FG and LG), the TFC increased with the drying temperature. High temperature treatments significantly decreased the TFC in all of the GS, with an up to 49% reduction in FG and 13% in LG after 100 °C treatment compared with the control temperature (30 °C). The TFC in the NGS and GS was not significantly impacted by thermal treatments for LR, which lacks the seed coat. On the basis of these findings, it is predicted that: (1) seed coats influence the TFC during the thermal processing of lentil NGS and GS; and (2) the types of flavonoids may vary among lentil cultivars, with varying thermal stabilities. Taking this into consideration, all major flavonoids were analyzed further and quantified using HPLC.

#### 3.3.2. Changes in Major Flavonoid Contents

Three major flavonoids (kaempferol, luteolin, and myricetin) were detected in the whole seeds of FG, LG, and LR [34]. The variations in the concentration of these compounds during germination or thermal processing were evaluated in the different cultivars of lentils (Table 1 and Table 2). Kaempferol, luteolin, and myricetin were detected in the whole seed of FG, while only kaempferol was found in LG and LR (Table 1). These findings are consistent with the previous results reported by Lee et al. [34]. However, the germination process led to various flavonoid variation patterns in the seeds of the different lentil cultivars, showing a decreased flavonoid content in FG and LG and an increased one in LR. Similar results were reported by Świeca et al. [31], who found that the germination process significantly reduced the contents of flavonoids such as catechin, quercetin, and luteolin, in lentil seeds. To confirm the effects of germination on the flavonoid contents in the different varieties of lentil, further analyses were performed using the endosperms and seed coats of FG and LG. The results of the analyses revealed that germination provoked an increase in the flavonoid contents in the endosperms of lentils, whereas it leads their reduction in the seed coats. Notably, luteolin and myricetin, which were not found in the FG NGS endosperm, were detected in the GS endosperm, at 32.18 and 31.67 μg/g DW, respectively. The kaempferol content in the LG endosperm also rose from 367.20 to 423.20 μg/g DW after germination. The increased enzyme activities associated with flavonoid production may be the cause of the peak in flavonoids during germination in the seed endosperm [35,36].

The effects of thermal processing on the flavonoid contents in the whole seed in all three cultivars of lentils, for both the NGS and GS, are shown in Table 2. Regardless of the cultivar, the NGS that had been exposed to high temperatures tended to have higher concentrations of each chemical compared to the treatments at 30 °C. In the dried NGS from FG, at 30 °C, the kaempferol concentration was 406.96 μg/g DW, luteolin was 107.11 μg/g DW, and myricetin was 131.14 μg/g DW. Each compound’s concentration increased after thermal processing, to 524.81, 120.84, and 134.04 μg/g DW, respectively. The kaempferol contents in LG and LR increased from 446.73 μg/g DW and 93.80 μg/g DW in the NGS after a 30 °C treatment to, respectively, 482.87 μg/g DW and 163.81 μg/g DW after treatments at higher temperatures. During heat processing, either the release of bound flavonoids or the acceleration of the extract ratio might account for this sort of increase [24,28,37]. The GS showed relatively low level of flavonoids in comparison with the NGS (Table 2). As mentioned above, this could be explained by the significant reduction in the flavonoid contents in the seed coat during germination (Table 1). The GS of FG and LG also exhibited an increase in flavonoid contents after thermal processing. For example, in the GS of FG, the kaempferol concentration reached 481.11 μg/g DW after the 100 °C treatment, luteolin was accumulated up to 94.12 μg/g DW after the 80 °C treatment, and myricetin reached up to 131.14 μg/g DW after the 80 °C treatment. Moreover, the highest kaempferol content was observed in the GS of LG at 100 °C. However, the thermal treatment reduced the kaempferol content in the GS from the LR (Table 2). These different observations on the effects of thermal treatments on different cultivars of lentil were tentatively explained by their respective seed compositions. The FG and LG, which possess hard and strong seeds coats, potentially had the ability to delay the heat transfer in the seeds. Meanwhile, due to the high water content in the GS of LR (without a seed coat), the thermal energy transfer was accelerated and led to a faster degradation of flavonoids.

### 3.4. Changes in Antioxidant Activity 

#### 3.4.1. The Effects of Germination on Antioxidant Activities Variation in Three Different Cultivars of Lentils

**Figure 4:**
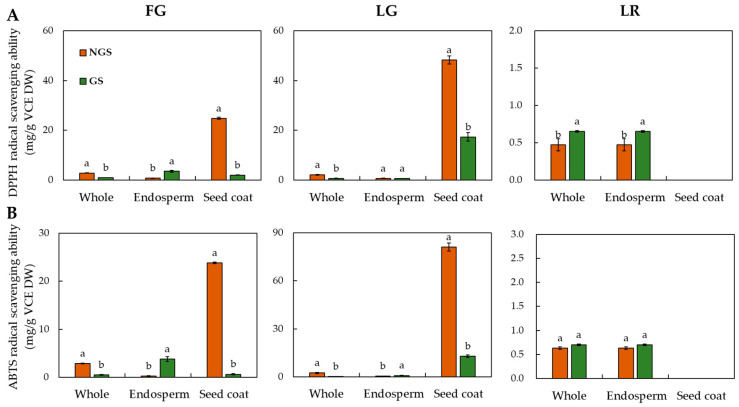
Changes in (**A**) DPPH and (**B**) ABTS radical scavenging ability of different cultivars of lentils during germination. Different letters indicate the significant difference in Tukey’s studentized test at *p* < 0.05.

The effects of germination on the DPPH and ABTS radical scavenging abilities in the different cultivars of lentil are shown in Figure 4. It was observed that the DPPH radical scavenging activities varied in the lentil cultivars (Figure 4A). The AA in the whole seeds (with seed coat) of the FG NGS was 2.83 mg/g VCE DW. For the LG NGS, it was 2.13 mg/g VCE DW, which was higher than the DPPH radical scavenging activity observed in the LR (without seed coat) NGS (0.48 mg/g VCE DW). These results were consistent with the ones reported by Lee et al. [34], who stated that lentil cultivars with seed coats exhibit higher DPPH radical scavenging ability than the ones without. The maximum DPPH radical scavenging activities for FG and LG were found in the seed coat and were, respectively, 28.84 and 48.20 mg/g VCE DW. Moreover, similar DPPH radical scavenging activities were observed in both the endosperms and the whole seeds of the NGS of these two lentil cultivars. However, the germination process significantly decreased the DPPH radical scavenging activity in the whole seeds of FG and LG, which were reduced by approximately 64% in the GS in comparison with the NGS. On the other hand, the DPPH radical scavenging activity in the GS from LR was significantly higher than in the NGS. The lower AA of the GS might be caused by a notable drop in the DPPH radical scavenging activity in the seed coat of both the FG and LG cultivars. Additionally, the FG endosperm’s ability to scavenge DPPH radicals was greatly enhanced during germination; however, this was not the case in the LG endosperm. The ABTS radical scavenging ability (Figure 4B) from the NGS of FG, LG, and LR were 2.86, 2.44, and 0.64 mg/g VCE DW, respectively. Similarly to the DPPH radical scavenging ability results, the seed coats exhibited much higher DPPH radical scavenging ability than the endosperms of FG and LG (Figure 4). These results are consistent with the results of numerous studies reporting that seed coats usually exhibit more AA than the endosperms/cotyledons in legume, such as soybean and common bean [26,38]. The germination process also decreased the ABTS radical scavenging ability of the whole seed in FG and LG. The significantly reduced ABTS radical scavenging ability of the seeds coats might be responsible for these results, as the AA of the endosperms were significantly increased (Figure 4B). In crops such as mung bean, soybean, radish, buckwheat, etc., the positive effects of germination on AA have been extensively reported [11,29,30]. The rise in AA during germination is likely related to the quantitative increase in antioxidants such as polyphenols. Nevertheless, it has been reported that the germination process decreased the AA of lentils compared to raw seeds, especially concerning the radical scavenging activities [31]. It is noteworthy that, whereas germination reduced the amount of AA in the seed coat, it provoked a rise in AA in the endosperm. In addition, germination considerably improved the ABTS radical scavenging ability of LG but not of LR, in contrast to the variations of DPPH radical scavenging ability in the endosperm of LG and LR. DPPH radicals tend to react with hydrophobic compounds, while ABTS radicals tend to react with both lipophilic and hydrophilic compounds [39]. Thus, these results might be explained by the antioxidant features and the quantity of GS, which depends on many factors, such as the legume cultivar.

#### 3.4.2. The Effects of Thermal Processing on Antioxidant Activities Variation in Different Cultivar of Lentils 

**Figure 5:**
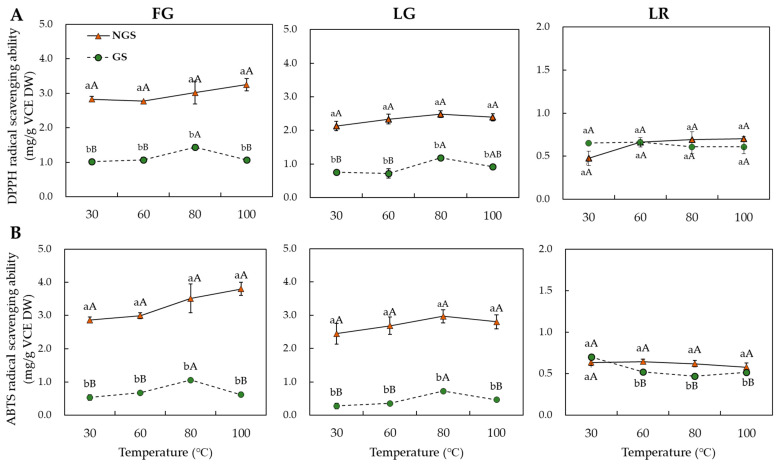
Changes in (**A**) DPPH, and (**B**) ABTS radical scavenging activities of different cultivars of lentil NGS and GS during thermal processing. Different lowercase letters indicate significant differences (Tukey’s studentized test at *p* < 0.05) between NGS and GS, and different capital letters indicate significant differences (Tukey’s studentized test at *p* < 0.05) between lentils treated at different temperatures.

The effects of thermal processing on the DPPH and ABTS radical scavenging activities in the different lentil cultivars are shown in Figure 5. Regardless of the lentil cultivars, there was no significant variation in the DPPH radical scavenging activity of the various temperature-treated lentil NGS, which were 2.77–3.25 mg/g VCE DW in FG, 2.1–2.48 mg/g VCE DW in LG, and 0.48–0.70 mg/g VCE DW in LR. However, the GS from the FG treated at 80 °C (1.44 mg/g VCE DW) and the GS from the LG treated at 80 °C (1.18 mg/g VCE DW) had significantly higher DPPH radical scavenging abilities than the other treatments. Nevertheless, there was no significant difference in the DPPH radical scavenging ability observed between each temperature treatment in the GS from LR. In terms of the ABTS radical scavenging ability (Figure 5B), the NGS from the FG and LG exhibited significantly higher values than the GS, regardless of the thermal treatment. Similarly to the variation pattern in the DPPH radical scavenging ability, thermal treatment did not affect the ABTS radical scavenging ability in the NGS of FG and LG cultivars, whereas it did in the GS. The strong seed coats in the NGS of FG and LG potentially reduce the heat transfer efficiency. Meanwhile, due to the high water content of the GS of FG and LG, the thermal energy transfer was accelerated, which led to the release of antioxidants [28], which may be an explanation for the significantly increased AA in the seeds at 80 °C. However, high temperature treatments also cause the degradation of antioxidants, such as vitamins and phenolics [28,40], which eventually leads to a reduction in AA. In the GS from LR, it was shown that the heat treatments drastically decreased the ABTS radical scavenging ability, while maintaining the DPPH radical scavenging ability at a level comparable to the NGS. As we mentioned in Section 3.4.1, while the ABTS radicals interact with both lipophilic and hydrophilic molecules, the DPPH radicals only react with hydrophobic compounds. These results suggested that the thermal processing might have affected the hydrophilic compounds in the germinated lentil seeds.

The correlation coefficient analysis showed that most of the values were highly correlated to each other, especially values exhibiting over 0.833 with *p* < 0.001 between TPC and AA (Table 3 and Table 4).

## 4. Conclusions

This study investigated the effects of thermal processing on the color and antioxidants of the NGS and GS of three different cultivars of lentil, including two cultivars with seed coats, FG and LG, and one cultivar without a seed coat, LR. The germination process made the seeds relatively lighter and reduced their redness and yellowness, regardless of the temperature changes. Regarding the antioxidant properties of the seeds, the NGS of FG and LG exhibited higher AA than those of LR. A further study proved that this difference was mainly due the existence of seed coats in the cultivars FG and LG. Moreover, the seed coats showed significantly higher AA than the endosperms. The germination process significantly decreased the AA in the whole seeds of FG and LG. As the AA values in the endosperms of the germinated seeds tended to increase in both cultivars, it has been concluded that the decrease in AA in the GS of FG and LG might be due to the reduction in antioxidants in the seed coat. Thermal processing at different temperatures did not significantly impact the seeds properties, regardless of the cultivar and germination state. However, when the processing temperature was increased to 80 °C, the AA gradually increased and peaked. The AA decreased at 100 °C in the GS of the three lentil cultivars. All of the major flavonoid components, TPC, TFC, and AA, exhibited high coefficient values in the correlation coefficient study. It can be concluded that, firstly, although the seed coat is an important source of antioxidants in lentil seeds, their contents decrease significantly after germination. However, germination tends to increase the antioxidants in all of the cultivars of lentil endosperm. Secondly, the effects of temperature on the contents of antioxidants in both the GS and NGS were not significant. Therefore, further research should be focused on screening lentil varieties suitable for germination and on regulating the germination process to increase the antioxidant content of lentil.

## Figures and Tables

**Table 1:** The flavonoid content (μg/g DW) variations in three cultivars of lentil NGS and GS.

		FG			LG			LR		
		Kaempferol	Luteolin	Myricetin	Kaempferol	Luteolin	Myricetin	Kaempferol	Luteolin	Myricetin
Whole	NGS	406.96 ± 15.71 b	107.11 ± 14.71 b	131.14 ± 17.25 b	446.73 ± 8.66 ab	N.D.	N.D.	93.80 ± 3.00 a	N.D.	N.D.
	GS	357.50 ± 38.29 b	66.20 ± 4.18 bc	78.53 ± 11.15 c	398.39 ± 47.51 b	N.D.	N.D.	103.64 ± 13.73 a	N.D.	N.D.
Endosperm	NGS	354.97 ± 41.27 b	N.D.	N.D.	367.20 ± 12.93 b	N.D.	N.D.	93.80 ± 3.00 a	N.D.	N.D.
	GS	337.02 ± 8.53 c	32.18 ± 1.04 cd	31.67 ± 1.70 d	423.20 ± 26.63 b	N.D.	N.D.	103.64 ± 13.73 a	N.D.	N.D.
Seed coat	NGS	539.98 ± 17.31 a	441.47 ± 27.86 a	572.45 ± 6.55 a	537.11 ± 10.10 a	22.03 ± 5.14 a	8.67 ± 0.20 a	N.D.	N.D.	N.D.
	GS	146.34 ± 3.04 c	111.62 ± 7.04 b	125.74 ± 9.33 b	104.30 ± 6.69 c	12.31 ± 1.84 a	11.84 ± 3.39 a	N.D.	N.D.	N.D.

N.D. indicated not detected. Alphabetically letters within a column indicate significant difference in Tukey’s studentized test at *p* < 0.05.

**Table 2:** The effects of thermal processing on flavonoid content (μg/g DW) variations in three cultivars of whole lentil NGS and GS.

Temperature		FG			LG			LR		
		Kaempferol	Luteolin	Myricetin	Kaempferol	Luteolin	Myricetin	Kaempferol	Luteolin	Myricetin
30 °C	NGS	406.96 ± 15.71 ab	107.11 ± 14.71 a	131.14 ± 17.25 a	446.73 ± 8.66 a	N.D.	N.D.	93.80 ± 3.00 ab	N.D.	N.D.
	GS	357.50 ± 38.29 b	66.20 ± 4.18 a	78.53 ± 11.15 a	398.39 ± 47.51 a	N.D.	N.D.	103.64 ± 13.73 ab	N.D.	N.D.
60 °C	NGS	524.81 ± 40.68 a	123.32 ± 20.53 a	128.39 ± 15.26 a	375.78 ± 39.27 a	N.D.	N.D.	84.67 ± 1.07 ab	N.D.	N.D.
	GS	401.13 ± 29.19 ab	51.72 ± 8.01 a	70.77 ± 7.50 a	365.51 ± 20.28 a	N.D.	N.D.	71.57 ± 1.90 ab	N.D.	N.D.
80 °C	NGS	480.27 ± 17.73 ab	120.84 ± 23.49 a	132.16 ± 19.88 a	482.87 ± 22.69 a	N.D.	N.D.	153.35 ± 48.84 ab	N.D.	N.D.
	GS	453.68 ± 15.07 ab	94.12 ± 2.01 a	116.12 ± 9.37 a	427.26 ± 7.73 a	N.D.	N.D.	64.41 ± 5.58 b	N.D.	N.D.
100 °C	NGS	444.16 ± 26.49 ab	115.16 ± 30.45 a	134.04 ± 32.73 a	461.55 ± 28.74 a	N.D.	N.D.	153.35 ± 48.84 ab	N.D.	N.D.
	GS	481.11 ± 29.50 ab	66.83 ± 14.95 a	74.37 ± 7.93 a	430.85 ± 57.39 a	N.D.	N.D.	163.81 ± 23.65 a	N.D.	N.D.

N.D. indicated not detected. Alphabetically letters within a column indicate significant difference in Tukey’s studentized test at *p* < 0.05.

**Table 3:** Correlation coefficient analysis between polyphenols, and antioxidant activities in three cultivars of lentil during seed germination.

	TPC	TFC	Kaempferol	Luteolin	Myricetin	DPPH	ABTS
TPC	1						
TFC	0.861 ***	1					
Kaempferol	0.508 ***	0.514 ***	1				
Luteolin	0.507 ***	0.498 ***	0.348 *	1			
Myricetin	0.490 ***	0.480 ***	0.353 *	0.993 ***	1		
DPPH	0.958 ***	0.785 ***	0.405 **	0.326 *	0.306 *	1	
ABTS	0.926 ***	0.778 ***	0.434 **	0.168 ^ns^	0.142 ^ns^	0.963 ***	1

Values marked with asterisks indicate significance by Pearson’s correlation analysis. * indicates *p* < 0.05, ** indicates *p* < 0.01, *** indicates *p* < 0.001, and ns indicates no significance.

**Table 4:** Correlation coefficient analysis between polyphenols, and antioxidant activities in three cultivars of lentil during thermal processing.

	TPC	TFC	Kaempferol	Luteolin	Myricetin	DPPH	ABTS
TPC	1						
TFC	0.744 ***	1					
Kaempferol	0.862 ***	0.663 ***	1				
Luteolin	0.587 ***	0.135 ^ns^	0.501 ***	1			
Myricetin	0.589 ***	0.149 ^ns^	0.502 ***	0.991 ***	1		
DPPH	0.904 ***	0.604 ***	0.666 ***	0.594 ***	0.590 ***	1	
ABTS	0.833 ***	0.560 ***	0.546 ***	0.507 ***	0.498 ***	0.973 ***	1

Values marked with asterisks indicate significance by Pearson’s correlation analysis. *** indicates *p* < 0.001, and ns indicates no significance.

## Data Availability

Not applicable.

## Abbreviations

NGS	Nongerminated Seeds
GS	Germinated Seeds
AA	Antioxidant Activity
FG	French Green
LG	Lentil Green
LR	Lentil Red
TPC	Total Phenolic Content
TFC	Total Flavonoid Content
